# Mobile phones improve antenatal care attendance in Zanzibar: a cluster randomized controlled trial

**DOI:** 10.1186/1471-2393-14-29

**Published:** 2014-01-17

**Authors:** Stine Lund, Birgitte B Nielsen, Maryam Hemed, Ida M Boas, Azzah Said, Khadija Said, Mkoko H Makungu, Vibeke Rasch

**Affiliations:** 1 Department of International Health, Immunology and Microbiology, University of Copenhagen, Blegdamsvej 3, 2200 Copenhagen, Denmark; 2 Department of Obstetrics and Gynaecology, Aarhus University Hospital, Aarhus, Denmark; 3 Ministry of Health, Revolutionary Government of Zanzibar, Stonetown, Zanzibar, Tanzania; 4 Department of Obstetrics and Gynaecology, Odense University Hospital, Odense, Denmark

**Keywords:** Antenatal care, Maternal health, Neonatal health, Mobile phones, mHealth, Zanzibar

## Abstract

**Background:**

Applying mobile phones in healthcare is increasingly prioritized to strengthen healthcare systems. Antenatal care has the potential to reduce maternal morbidity and improve newborns’ survival but this benefit may not be realized in sub-Saharan Africa where the attendance and quality of care is declining. We evaluated the association between a mobile phone intervention and antenatal care in a resource-limited setting. We aimed to assess antenatal care in a comprehensive way taking into consideration utilisation of antenatal care as well as content and timing of interventions during pregnancy.

**Methods:**

This study was an open label pragmatic cluster-randomised controlled trial with primary healthcare facilities in Zanzibar as the unit of randomisation. 2550 pregnant women (1311 interventions and 1239 controls) who attended antenatal care at selected primary healthcare facilities were included at their first antenatal care visit and followed until 42 days after delivery. 24 primary health care facilities in six districts were randomized to either mobile phone intervention or standard care. The intervention consisted of a mobile phone text-message and voucher component. Primary outcome measure was four or more antenatal care visits during pregnancy. Secondary outcome measures were tetanus vaccination, preventive treatment for malaria, gestational age at last antenatal care visit, and antepartum referral.

**Results:**

The mobile phone intervention was associated with an increase in antenatal care attendance. In the intervention group 44% of the women received four or more antenatal care visits versus 31% in the control group (OR, 2.39; 95% CI, 1.03-5.55). There was a trend towards improved timing and quality of antenatal care services across all secondary outcome measures although not statistically significant.

**Conclusions:**

The wired mothers’ mobile phone intervention significantly increased the proportion of women receiving the recommended four antenatal care visits during pregnancy and there was a trend towards improved quality of care with more women receiving preventive health services, more women attending antenatal care late in pregnancy and more women with antepartum complications identified and referred. Mobile phone applications may contribute towards improved maternal and newborn health and should be considered by policy makers in resource-limited settings.

**Trial registration:**

ClinicalTrials.gov, NCT01821222.

## Background

With more than 600 million mobile phone users in Africa, applying mobile phones in healthcare, mHealth, is increasingly prioritized to strengthen healthcare systems [1,2]. Targeting mobile phone interventions to women in low income countries is appealing owing to the potential of empowering women to make informed choices in relation to their health. The interest to use mobile phones to promote reproductive health is not yet reflected in research evidence for effectiveness. We know of no other cluster-randomized controlled trial that has assessed the use of a mobile phone intervention to improve access to essential reproductive health services in a resource-limited setting.

Recent evidence indicates steady progress towards the achievement of Millennium Development Goals (MDG) 4: reduce child mortality, and MDG 5: improve maternal health. However, with approximately 270.000 maternal and 3 million annual neonatal deaths, reduction of maternal and neonatal mortality remains a global challenge [3,4]. Further, it is estimated that at least 2.65 million stillbirths occur worldwide, many of them due to preventable causes related to poor maternal health and most of these happens in low and middle-income countries [5]. Antenatal care has the potential to reduce maternal morbidity and improve newborns’ health [6,7]. It provides pregnant women with a broad range of health promotion and preventive health services and is important in identifying risk factors for adverse pregnancy outcomes. Inadequate antenatal care is related to poor pregnancy outcome and women who seek antenatal care late with few visits are less likely to be assisted during delivery by a skilled attendant [8]. With an emphasis on quality over quantity the revised Focused Antenatal Care model of the World Health Organization (WHO) recommends at least four antenatal care visits for uncomplicated pregnancies with the first visit starting before 16 weeks of gestation [9]. However, lack of relevant and high quality antenatal care is a major concern for many pregnant women in sub-Saharan Africa [10]. According to the 2012 MDG report the proportion of women attending antenatal care four times or more by any provider during pregnancy decreased in sub-Saharan Africa from 50% in 1990 to 46% in 2010 [11].

This article presents results from a cluster-randomized controlled trial aimed to evaluate the association between a mobile phone intervention named “wired mothers” and antenatal care in Zanzibar. We assessed the hypothesis that the *Wired Mothers* intervention can increase antenatal care attendance as well as improve content and timing of antenatal care services provided to individual women.

## Methods

*Wired mothers* is a pragmatic cluster-randomised controlled trial with the primary healthcare facility as the unit of randomisation. The study took place from March 2009 to March 2010 in Zanzibar, United Republic of Tanzania. The study design and intervention have previously been described in detail [12]. The national ethical committee, Research Council of Zanzibar, approved the study protocol on 27^th^ January 2009 with reference number 23. The nature and purposes of the study was summarized in a consent form in the local language Swahili that was presented to all women eligible for inclusion in the study. All participating women provided informed consent either by signature or fingerprint. Women were free to drop up of the study at any time without a change in the quality of care provided to them.

### Setting

Zanzibar is a semi-autonomous part of the United Republic of Tanzania and consists of numerous small islands and two large ones. The wired mothers study took place on the island of Unguja. The island has six districts with 80 healthcare facilities. Of the six districts, two are urban and four rural.

### Participants

**Figure 1:**
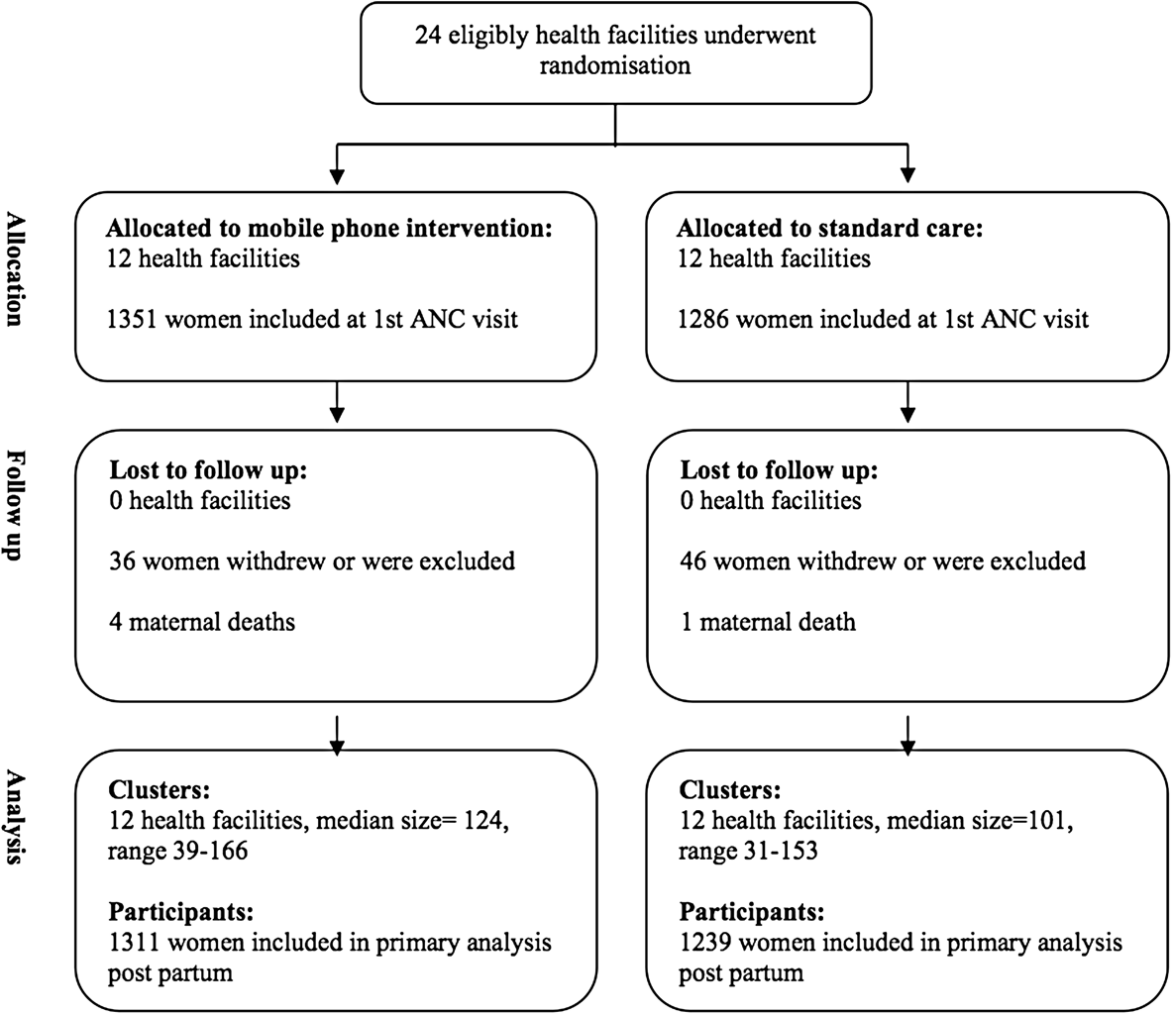
Procedures for selection of study population.

As this is a cluster design, eligibility criteria apply to both the primary healthcare facility and individual levels of analysis. In each district, the four primary healthcare facilities with highest level of antenatal care attendance in the preceding year and staffed with at least one midwife were included. At individual level, the study included 2550 women distributed in the 24 primary healthcare facilities (Figure 1). Women who attended antenatal care at selected healthcare facilities, were included on their first antenatal care visit and followed until 42 days after delivery. Women were eligible for study participation irrespective of their mobile phone ownership and literacy status. The terminology “wired mothers” was used to describe women linked to the health system by use of a mobile phone intervention throughout their pregnancy and postpartum period.

### Intervention

The wired mothers’ intervention consisted of an automated short messaging service (SMS) system providing wired mothers with unidirectional text messaging and a mobile phone voucher system providing the possibility of direct two-way communication between wired mothers and their primary health care providers. While only women with registered phone numbers received text messages, all women in the intervention group were given mobile phone vouchers to contact their local primary health care provider. The aim of the SMS component was to provide simple health education and appointment reminders to encourage attendance at routine antenatal care, skilled delivery attendance and postnatal care. A wired mothers software was developed to automatically generate and sent text messages to registered phone numbers throughout the pregnancy until six weeks after delivery. Based on the gestational age of the women at first antenatal care visit the wired mothers software creates an individual pregnancy time schedule. A welcome message was send at registration regardless of gestational age. Hereafter the content of messages varied depending on individual gestational age. The frequency was two messages per month before gestational week 36 and intensified till two per week from gestational week 36. The information required for the SMS software, gestational age, date and mobile phone number, was gathered during the first antenatal care visit and entered into the web based system. The registered phone numbers were either the women’s own phone or an access phone number of a relative who could relay the text messages. If the women could not provide a phone number she benefitted only from the mobile phone voucher component. The content of the messages were developed by a team of international researchers and local partners from the Ministry of Health in Zanzibar. Message content was standardised with neutral phrasing and provided as simple text in the local language of Swahili. In addition, primary health care facilities randomised for intervention and hospitals were provided with a mobile phone to improve timely referrals between different levels of the health system and to enable health workers in the periphery of the health system to consult patients with higher levels of care. To further improve access to emergency obstetric care, communication and referral links wired mothers were given a phone voucher with modest credit and a card with the phone number of her local primary health care provider allowing all wired mothers to communicate directly with primary health care providers.

### Outcomes

We evaluated the effect of a mobile phone intervention on two different outcomes. The present paper is concerned with antenatal care whereas another manuscript is concerned with skilled delivery attendance [12]. The primary outcome measure for antenatal care was the number of women receiving four or more antenatal care visits, and secondary outcome measures were quality of care indicators reflecting content and timing of antenatal care services according to the recommended antenatal care package for pregnant women in Zanzibar (Table 1), specifically: number of women receiving anti-tetanus vaccinations, preventive treatment for malaria, gestational age at last antenatal care visit, antepartum referrals and the timing of the mentioned services in gestational age [13].

**Table 1:** Recommended timing and content of antenatal visits

	**First visit**	**Second visit**	**Third visit**	**Fourth visit**
	<16 weeks	20–24 weeks	28–32 weeks	36–40 weeks
*Goal*	Confirm pregnancy and expected date of delivery	Assess maternal and fetal well-being	Assess maternal and fetal well-being	Assess maternal and fetal well-being
	Classify women for basic ANC or more specialized care	Exclude PIH and anaemia	Exclude PIH and anaemia and multiple pregnancies	Exclude PIH, anaemia, multiple pregnancies and malpresentation
	Screen, treat and give preventive measures	Give preventive measures	Give preventive measures	Give preventive measures
	Develop a birth and emergency plan	Review and modify birth and emergency plan	Review and modify birth and emergency plan	Review and modify birth and emergency plan
	Advice and counsel	Advice and counsel	Advice and counsel	Advice and counsel
*Screening and test*	Blood pressure	Blood pressure	Blood pressure	Blood pressure
	Haemoglobin	Haemoglobin	Haemoglobin	Haemoglobin
	Protenuria*	Protenuria*	Protenuria*	Protenuria*
	Bacteriuria**	Bacteriuria**	Bacteriuria**	Bacteriuria**
	HIV			
	Syphilis			
	Blood/Rh group**			
*Preventive measures*	Tetanus toxoid***		Tetanus toxoid***	
	Iron and folate	Iron and folate	Iron and folate	Iron and folate
		IPTp****	IPTp****	
		ARV if eligible	ARV if eligible	ARV if eligible

*Only nulliparous women, women with previous pre-eclampsia and women with diastolic blood pressure above 90. **Additional intervention for use in referral centres but not recommended as routine for resource-limited settings. *** TT1 at first antenatal care visit, TT2 at least four weeks after, TT3 at least six months later, TT4 at least 1 year later, TT5 at least one year later. Five doses are considered to give protection during the rest of the childbearing years. ****1^*st*^*dose gestational week 16–28, 2*^*nd*^*dose gestational week 28–40. There should be at least four weeks between doses. ANC = antenatal care, PIH = Pregnancy Induced Hypertension, IPTp = Intermittent Preventive Treatment in pregnancy.*

### Sample size calculation

Power calculations were made on the outcomes skilled birth attendance and antenatal care attendance and did not take into account the clustering effect. We started by enrolling antenatal care attendees during a three months period. According to the health management information system records from the previous year we could expect 1,400 women in the intervention group and 1,100 (80%) was estimated to complete a follow up interview 42 days postpartum whereas 1,720 women would be enrolled as non-wired mothers (control group) and an estimated 1,375 (80%) would be followed until 42 days post partum. To estimate whether this sample size was sufficient for detection of public health relevant effects of the intervention, we used data from the Tanzanian Demographic Health Survey (DHS 2005). With a 95% probability and a power of 90% 590 women (295 in each group) were necessary for showing an increase of a relevant size (10% increase in the number of women receiving four or more antenatal care visits). Hence, according to our power calculations, our proposed sample size was sufficient to document an effect of our intervention.

### Randomisation and masking

Primary healthcare facilities, stratified by district, were assigned by simple random allocation to either the mobile phone intervention or control group. Neither study participants nor clinic staff were masked because of the nature of the intervention requiring overt participation. Analysis accounted for within-cluster correlation of women cared for at the same facility. The average cluster size was 106 women: with a range of 26 to 146.

### Procedures and data collection

All enrolled women were offered standard maternal health services consisting of at least four antenatal care visits, skilled attendance at delivery and a postnatal visit within the first 48 hours for deliveries taking place outside health facilities [13]. Optimal conditions for provision of quality care in both intervention and control sites were ensured with the distribution of essential drugs for provision of antenatal care, electronic blood pressure meters, weighing scales, hemocues for measuring hemoglobin and urinalysis sticks. The selected primary health care facility staff also functioned as research assistants. Research assistants in intervention facilities received training on the mobile phone intervention. Each district was assigned a supervisor who visited all facilities once a week during the study period for quality control. Supervisors reported any encountered problems to the research team.

Demographic and covariate information were recorded with structured questionnaires at inclusion and six weeks after delivery. In between, all contacts with the health system were recorded at antenatal care, delivery and postnatal care visits. All enrolled women received an individual identity number and card. If the women did not return for the end-of-study interview the research assistant contacted them either by phone or directly. Radio announcements were also used to request women to provide the end-of-study interview. Double entry of data was performed in Epidata and transferred and validated in SPSS (version 20).

### Statistical analysis

Analyses were performed according to the intention-to-treat principle, and all available data were included in the analysis. Double entry of data was performed in Epidata and transferred and validated in SPSS (version 20) where all statistical analysis were conducted. The primary outcome was antenatal care attendance and logistic regression analysis was performed on the binary outcome of four or more antenatal care visits (yes or no) to assess the impact of the intervention. Because facilities rather than individual women were randomised, we used generalised estimating equations to account for within-cluster correlation in all statistical analysis.

The statistical model was developed initially including all variables shown in Table 2 as explanatory variables (including two-factor interactions) in a logistic regression analysis. The model was reduced by removing non significant confounders using backwards elimination. This resulted in a final model including age, literacy, gestational age at first antenatal care visit and intervention status. No interaction with intervention was identified. The logistic regression model was used to assess the primary as well as secondary outcomes. We also performed a Poisson loglinear analysis of continuous antenatal care visits to assess the intervention impact with mean number of antenatal care visits. Timing (in gestational age) of secondary outcomes was analysed using a linaer multiple regression model.

**Table 2:** Baseline characteristics of the study population. Values are numbers (%)

**Variable**	**Intervention**	**Control**
**Health facilities**		
No	12	12
**Participants**		
No of women	1311	1239
Age		
<19	107/1258 (9%)	118/1197 (10%)
20–24	310/1258 (25%)	307/1197 (26%)
25–29	371/1258 (29%)	309/1197 (26%)
30–34	248/1258 (20%)	259/1197 (22%)
35+	222/1258 (18%)	204/1197 (17%)
Literacy		
Can read very well	3601298 (28%)	384/1221 (31%)
Can read well	447/1298 (34%)	363/1221 (30%)
Can read some	178/1298 (14%)	174/1221 (14%)
Can read little	86/1298 (7%)	88/1221 (7%)
Cannot read	227/1298 (18%)	212/1221 (17%)
Education		
No	204/1278 (16%)	220/1200 (18%)
Primary	464/1278 (36%)	440/1200 (37%)
Secondary and above	569/1278 (45%)	503/1200 (42%)
Other	41/1278 (3%)	37/1200 (3%)
Mobile phone status		
Owns	494/1307 (38%)	439/1235 (36%)
Does not own	813/1307 (62%)	796/1235 (65%)
Residence status		
Rural	743/1311 (57%)	730/1239 (59%)
Urban	568/1311 (43%)	509/1239 (41%)
Parity		
Nullipara	264/1290 (21%)	233/1201 (19%)
1–2	428/1290 (33%)	356/1201 (30%)
3–4	292/1290 (23%)	297/1201 (25%)
5+	306/1290 (24%)	315/1201 (26%)
Gestational age at first antenatal care visit		
<16	256/1310 (20%)	329/1233 (27%)
17–26	930/1310 (71%)	814/1233 (66%)
27–35	121/1310 (9%)	87/1233 (7%)
36+	3/1310 (0%)	3/1233 (0%)
Complication last pregnancy according to mother		
Yes	89/1271 (7%)	122/1271 (11%)
No	918/1271 (72%)	811/1271 (70%)
Nullipara	264/1271 (21%)	233/1271 (20%)

Results were expressed as odds ratios (ORs) for primary and secondary binary outcomes and differences for linear multiple regression with 95% confidence intervals (95% CI). Statistical significance was defined as P < 0.05.

## Results

The sociodemographic characteristics of the intervention and control group were similar, with most women being housewives with limited education and literacy (Table 2). Thirty-seven percent of the women included in the study owned a mobile phone. The remaining had various degrees of access to mobile phones. Only 20% of the women in the intervention group and 27% in the control group attended their first antenatal care visit before gestational week 16 as recommended in national and international guidelines. Because the recruitment to the study was done at the first antenatal visit the timing of this visit could not be affected by the intervention. There was no difference between intervention and control groups by maternal risk factor such as young/old age, late antenatal care booking, nulliparity and previous pregnancy complication. Table 3 shows the characteristics of primary and secondary outcomes. Seven percent of intervention women attended antenatal care only once versus 18% of control women. Women who booked for antenatal care after gestational week 28, women below 19 years of age and illiterate were less likely to receive four or more antenatal care visits. Women who booked for antenatal care before gestational week 16, and those who were able to read well or very well were more likely to receive four or more antenatal care visits (data not shown).

**Table 3:** Characteristics of primary and secondary outcomes

	**Intervention**	**Control**
**Primary outcome**		
No of antenatal care visits		
1	92/1311 (7%)	222/1239 (18%)
2	222/1311 (17%)	258/1239 (21%)
3	423/1311 (32%)	374/1239 (30%)
4	380/1311 (29%)	234/1239 (19%)
>5	194/1311 (15%)	151/1239 (12%)
**Secondary outcomes**		
Tetanus vaccination of nullipara*		
TT1^1^	223/232 (96%)	195/208 (94%)
TT2^2^	155/232 (72%)	112/201 (56%)
Intermittent Preventive Treatment in pregnancy		
IPTp1	1191/1311 (91%)	1060/1239 (86%)
IPTp2	846/1311 (65%)	640/1239 (52%)
Gestational age at last antenatal care visit		
<16	17/1307 (1%)	44/1230 (4%)
17–26	140/1307 (11%)	254/1230 (21%)
27–35	784/1307 (60%)	684/1230 (56%)
36+	366/1307 (28%)	248/1230 (20%)
Antepartum referral	127/1311 (10%)	57/1239 (5%)

Data are n/N (%).*N = 264 intervention and 233 control.^*1*^*52 not eligible.*^*2*^*68 not eligible.*

The timing of antenatal care visits and preventive health services were similar in both the intervention and control groups (Table 4). Visit numbers one and two were later than recommended while visit numbers three and four were earlier. Tetanus vaccinations were given later than recommended while preventive treatment for malaria was administered within the recommended timeframe. The mean gestational age at last antenatal care visit was higher for intervention women than for control; both were however below the recommendation of last visit after gestational week 36. The mean gestational age at antepartum referral was 29.47 for intervention and 26.67 for control women.

**Table 4:** Timing in gestational age of primary and secondary outcomes

	**Intervention mean (+- SD)**	**Control mean (+- SD)**	Unadjusted difference* (95% CI)	Adjusted difference** (95% CI)
**Primary outcome**				
Weeks of gestation at antenatal care visit no				
1	20.71 (4.69)	20.10 (4.80)	0.68 (-0.39–1.74)	0.64 (-0.44–1.73)
2	26.13 (4·04)	25.92 (4.29)	0.20 (-0.45–0.86)	0.13 (-0.51–0.77)
3	30.27 (3·50)	30.41 (3.88)	-0.32 (-1.31–0.67)	-0.37 (-1.34–0.61)
4	33.39 (3·05)	32.79 (3.14)	0.21 (-0.83–1.25)	0.29 (-0.72–1.30)
5	35.45 (2·45)	34.77 (2.37)	0.53 (-0.46–1.51)	0.45 (-0.57–1.47)
**Secondary outcomes**				
Weeks of gestation at tetatnus vaccination of nullipara***				
TT1	20.28 (5.08)	19.46 (5.15)	0.90 (-0 · 39–2.18)	-0.05 (-0.94–0.85)
TT2	25.53 (4.05)	25.42 (4.78)	0.07 (-1.10–1.24)	-0.84 (-1.93–0.26)
Weeks of gestation at Intermittent Preventive Treatment in pregnancy				
IPTp1	23.75 (3.48)	22.93 (3.84)	0.80 (0.06-1.54)	0.83 (0.06–1.59)
IPTp2	29.33 (3.25)	28.90 (3.48)	0.59 (-0.49–1.66)	0.59 (-0.48–1.65)
Weeks of gestation at last antenatal care visit	31.81 (4.90)	30.15 (5.86)	1.41 (-0.21–3.02)	1.38 (-0.23–2.99)
Weeks of gestation at antepartum referral	29.47 (6.38)	26.67 (8.06)	2.90 (-0.58–6.38)	2.67 (-0.54–5.89)

*Adjusted for within cluster effect.**Adjusted for significant variables associated with antenatal care attendance and within cluster effect.***N = 264 intervention and 233 control.^*1*^*52 not eligible.*^*2*^*68 not eligible. SD Standard Deviation.*

More women in the intervention group (44%) received the recommended four or more antenatal care visits than in the control group (31%) (Table 5). The odds for receiving four or more antenatal care visits were more than double for women benefiting from the mobile phone intervention (OR, 2.39; 95% CI, 1.03-5.55). There was a trend towards favorable intervention association across all secondary outcome measures although not statistically significant. In the intervention group 72% of nullipara women received two doses of tetanus vaccination versus 56% in the control group (OR, 1.62; 95% CI, 0.81-3.26) and 65% received two doses of preventive malaria treatment versus 52% in the control group (OR, 1.97; 95% CI, 0.98-3.94). More intervention women (28%) had their last antenatal care visit after gestational week 36 compared to control women (20%) (OR, 1.48; CI 0.89-2.45), and 10% intervention women were referred during the antepartum period compared to 5% control women (OR, 1.66; 95% CI, 0.68-4.06). Overall, women in the intervention group received 16% more antenatal care visits than the control group. 385 women, 30%, called their health provider. Calls were made both in case of emergencies and for non-emergencies. The majority, 59%, of intervention women stated that receiving text messages influenced the number of times they attended antenatal care. In addition, 71% felt that the educational messages helped them in various areas including learning about danger signs in pregnancy and feeling that the health system cared for them.

**Table 5:** Association between mobile phone intervention and primary and secondary outcomes

	**Intervention**	**Control**	Unadjusted OR* (95% CI)	Adjusted OR** (95% CI)
**Primary outcome**				
Four or more antenatal care visits	574/1311 (44%)	385/1239 (31%)	1.54 (0.80–2.96)	2.39 (1.03–5.55)
**Secondary outcomes**				
Tetatnus vaccination of nullipara***				
TT1^1^	223/232 (96%)	195/208 (94%)	1.38 (0.39–4.87)	1.58 (0.41–6.01)
TT2^2^	155/232 (72%)	112/201 (56%)	1.67 (0.84–3.33)	1.62 (0.81–3.26)
Intermittent Preventive Treatment in pregnancy				
IPTp1	1191/1311 (91%)	1060/1239 (86%)	1.78 (0.49–6.52)	1.10 (0.35–3.43)
IPTp2	846/1311 (65%)	640/1239 (52%)	1.69 (0.82–3.48)	1.97 (0.98–3.94)
Gestational age 36 or more at last antenatal care visit	366/1307 (28%)	248/1230 (20%)	1.45 (0.88–2.37)	1.48 (0.89–2.45)
Antepartum referral	127/1311 (10%)	57/1239 (5%)	1.58 (0.61–4.09)	1.66 (0.68–4.06)

*Adjusted for within cluster effect.**Adjusted for significant variables associated with antenatal care attendance, within cluster effect.***N = 264 intervention and 233 control.^*1*^*52 not eligible.*^*2*^*68 not eligible.*

## Discussion

Our findings showed that a simple mobile phone intervention improved antenatal care attendance. Women in the intervention group had more than double odds for attending four or more antenatal care visits as recommended in national and international guidelines. There was also a trend towards favorable intervention association across secondary outcome measures such as tetanus vaccination, preventive treatment for malaria, gestational age at last antenatal care visit, and antepartum referral although not statistically significant.

We used simple mobile phone technology to address irregular attendance to essential reproductive health services in low-income countries. Evidence of interventions that effectively improve utilization of antenatal care services is particularly important in sub-Saharan Africa which has the lowest antenatal care attendance compared to other regions. Regular attendance to antenatal care throughout the pregnancy is important to identify complications of pregnancy and improve pregnancy outcomes for mother and child [6]. In our study there was a trend towards more antepartum referrals among the intervention group, indicating that more women with complications were being identified and treated. Worldwide, fewer newborns are dying but they account for a higher share of child deaths and estimates suggest that 14% of all deaths amongst children under five are due to preterm birth complications [3]. As there is an association between few antenatal care visits and a subsequent preterm birth, regular attendance to antenatal care is essential to improve child survival [14].

The weak relationship between antenatal care use and maternal and newborn survival has motivated an increased focus on content and quality of care provided rather than mere antenatal care attendance [15]. We therefore aimed to assess antenatal care in a more comprehensive way considering not only utilization but also content and timing of interventions during pregnancy. A recent publication on quality of antenatal care in Zambia similarly suggest that evaluating the level of antenatal care provision at heath facilities is an efficient way to detect deficiencies in quality of care [16]. Our results suggest that mobile phone interventions can improve quality of care by creating awareness on the demand side of service delivery. We found a trend towards more women receiving preventive health interventions but the timing of services was not significantly improved by the intervention. The effectiveness of antenatal care interventions is linked to its quality. Nyamtema et al. found that in rural Tanzania 20% of severe maternal morbidities were attributed to substandard antenatal care which indicates that a significant proportion of adverse pregnancy outcome could be reduced by improving this programme [17]. Women can receive the same content of care in a different number of visits and more comprehensive indices are needed to assess quality of the antenatal care provided. There are few studies on quality of antenatal care and the existing tools to capture dimensions of quality of antenatal care have been developed for high-income settings and we found them unsuitable for application in a low-income countries context. For instance, the Content and Timing of care in Pregnancy (CTP) tool includes routine ultrasound and measurement of blood glucose, services that are neither readily available nor included in the core antenatal care package recommended for low-income countries [18]. There are attempts to develop tools tailored for resource-limited settings but the framework for assessment of provision of quality antenatal care in resource limited settings remains weak [16,19].

Our findings are in line with the recent reviews concluding that there is moderate evidence that mobile phone text message reminders for health care appointments are more effective than no reminders and that text messaging can result in positive health behavior change [20-22]. In 2013 Free et. al. assessed the effectiveness of mobile technology interventions delivered to health care consumers and found that text messaging interventions increased adherence to ART and smoking cessation and stated that high quality adequately powered trials are required to eveluate effects of objective outcomes [20]. The most robust evidence is on the effectiveness of Short Message Service reminders increasing attendance to health care services. Two recent reviews found that SMS reminders increase the likelihood of attending clinic appointments [21,22]. These reviews however included only a limited number of randomized controlled trials and all were from high and middle income countries. Hence, external validity for the developing world is limited. There is an increasing number of mHealth pilot projects in low-income countries, most of which have a disease specific focus on HIV/AIDS [23,24]. The 2012 Lancet report of technologies for global health identified only nine randomised controlled trials for mHealth in low-income countries and the evidence for effectiveness remains weak [2,23,24]. Within the use of mobile phones to improve reproductive health studies primarily indicate potential to expediting emergency obstetric referrals and improve knowledge and awareness [25-27]. Our study has produced evidence of increased skilled delivery attendance amongst urban women benefiting from the wired mothers intervention (OR, 5.73; 95% CI, 1.51-21.81) [12]. Most of the women in our study felt that the intervention influenced their health seeking behavior and satisfaction with the service which is in line with other studies finding mobile phone applications for reproductive health well accepted by women and health workers [28-30]. Further studies of interventions similar to ours are currently taking place in Ghana, for instance, and the evidence base is growing fast [31].

There were limitations to our study. We chose a pragmatic approach and randomized health facilities rather than individuals to avoid a spillover effect from the intervention to the control group. Cluster design has been used in similar antenatal care trials [9,32]. We included women regardless of mobile phone ownership and literacy status primarily because we did not feel it ethical to limit access to emergency obstetric care based on mobile ownership. In addition, there is evidence of heterogeneous mobile phone ownership and usage patterns in sub-Saharan Africa and we believed that the SMS content would reach some women without own registered phone numbers [33]. We chose to provide health workers in intervention facilities with mobile phones to ensure equal access to health providers. We introduced the mobile phone as a professional working tool because we did not find it reasonable to ask health providers to provide their telephone numbers to clients and it allowed us to set up guidelines for use such as always keeping the phone charged and on. Our results indicate that linkages were improved between Wired Mothers and their health providers with almost one third of intervention women calling their local midwife at some point in pregnancy. The ability of pregnant women to call their local midwife was during the trial based on a voucher system, which is possible not perceived feasible at a larger scale than this study. Other solutions could be considered such as using a central toll free number, which will redirect calls to local midwifes. The follow-up rate in our study was high which may be attributed to the use of regular health workers as research assistants with personal local knowledge of included *Wired Mothers*. It was however demanding in terms of supervision to ensure data quality and could affect the quality of practices provided to women. We did not assess health provider’s skills and knowledge and hence cannot be sure that particularly secondary outcomes measures were confounded by intervention sites health provider knowledge. However, health providers in intervention facilities did not have access to the educational messages send to Wired Mothers and the study design should protect against both confounders.

We aimed towards a frugal innovation taking advantage of the ubiquity of mobile phones and the technical solution was produced in Tanzania at low cost. Yet future improvements should include special attention to the illiterate, such as using voice SMS and the use of women groups as the community entry point to reach the most vulnerable women who do not have access to mobile phones and do not attend antenatal care even once. While this study demonstrates that the wired mothers intervention can improve the number of repeat antenatal care visits it does not, in its present form, target the problem of late booking for antenatal care. Further research should examine if inclusion into the wired mothers software at community level will improve the number of women attending their first antenatal care visit before gestational week 16 as recommended by WHO. For instance, a study from Kenya suggests that pregnancy case finding can be performed at community level by village elders using mobile phones [34].

The policy implication of this study is that mobile phone interventions can improve utilization of antenatal care services, which is essential for maternal and newborn health. Although not statistically significant our findings also point in the direction of mobile phone interventions being effective in improving adherence to antenatal care schedules and quality of care. Only nine of 137 developing countries are likely to achieve both the MDG 4 and 5 targets by 2015 [4]. We suggest that mHealth applications can assist in achieving the MDG target indicators and more importantly reduce maternal morbidity and improve newborn survival. Policy makers should consider using mobile phone applications to improve attendance and quality of care of essential reproductive health services. Furthermore, our study indicates that measurement of development after the MDGs should include use of beneficial technologies such as mobile phones.

## Conclusions

The wired mothers’ mobile phone intervention significantly increased the proportion of women receiving the recommended four antenatal care visits during pregnancy and there was a trend towards more women receiving preventive health services, more women attending antenatal care late in pregnancy and more women with antepartum complications identified and referred. Overall there is limited evidence on the effects of mobile phone text message reminders for appointment attendance and further high-quality research is required to draw more robust conclusions, particularly for developing countries within the field of sexual and reproductive health. This study is a contribution towards evidence-based approaches to make pregnancy and childbirth a safe event for both mother and child, and towards the achievement of the MDG 5 target indicator of antenatal care attendance.

## Abbreviations

ANC: Antenatal care; IPTp: Intermittent preventive treatment in pregnancy; MDG: Millennium development goal; PIH: Pregnancy induced hypertension; SMS: Short messaging service; WHO: World Health Organization.

## Competing interests

All authors declare that they have no competing interests.

## Authors’ contribution

All authors were involved in the development of the study design and implementation plan. SL was the principal investigator for the study and SL and VR are the guarantors. SL, MH, IMB, AS, KS, MHM were responsible for implementation of the study and BBN and VR for overall supervision. SL and VR did the quantitative analysis. SL wrote the initial draft of the paper. All authors critically reviewed the manuscript and approved the final version.

## Pre-publication history

The pre-publication history for this paper can be accessed here:

http://www.biomedcentral.com/1471-2393/14/29/prepub
